# Supplementary material for: Retrosynthesis prediction using an end-to-end graph generative architecture for molecular graph editing
Source: Nat Commun. 2023 May 25;14:3009. doi: 10.1038/s41467-023-38851-5 (PMC10209957; doi:10.1038/s41467-023-38851-5)
Supplement: Supplementary file 1 — Supplementary Information [file 41467_2023_38851_MOESM1_ESM.pdf]

# Supplementary Information

# Retrosynthesis prediction using an end-to-end graph generative architecture for molecular graph editing

Weihe Zhong[1,2], Ziduo Yang[1], Calvin Yu-Chian Chen[1,3,4*]

[1] Artificial Intelligence Medical Research Center, School of Intelligent Systems Engineering, Shenzhen Campus of Sun Yat-sen University, Shenzhen 518107, China

[2] School of Biomedical Engineering, Shenzhen Campus of Sun Yat-sen University, Shenzhen 518107, China

[3] Department of Medical Research, China Medical University Hospital, Taichung 40447, Taiwan

[4] Department of Bioinformatics and Medical Engineering, Asia University, Taichung 41354, Taiwan

## Supplementary Figures

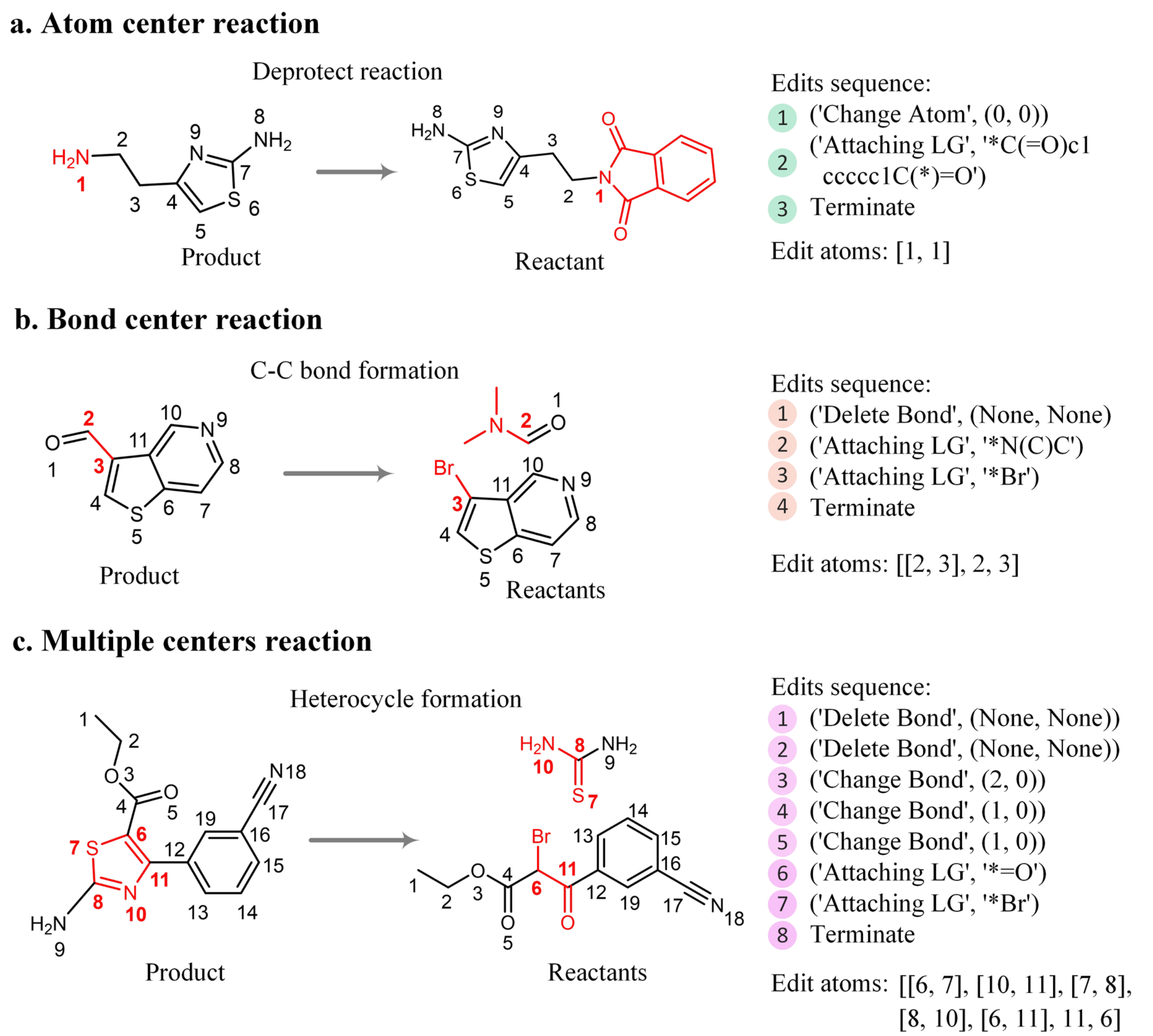


**Supplementary Fig. 1**. **The examples of derived edits sequence in retro-reactions. a** Atom center reaction, **b** Bond center reaction, and **c** Multiple centers reaction. The two numbers in brackets of atom edit represent the number of hydrogen and the chiral type to be changed, and the two numbers in brackets of bond edit indicate the bond type and the bond stereo configuration to be changed. The changes in the reaction are highlighted in red.

**a. Amino protection reaction**

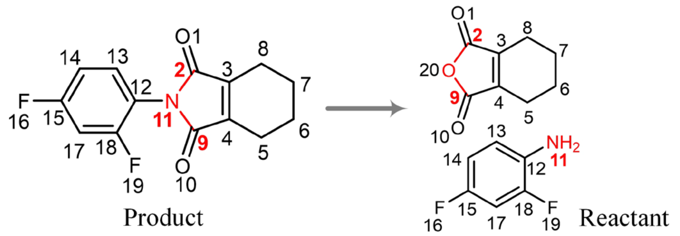


Incorrect edits sequence:

1. ('Delete Bond', (None, None))
2. ('Delete Bond', (None, None))
3. Terminate

Edit atoms: [[9, 11], [2, 11]]

**b. Aldehyde deprotection reaction**

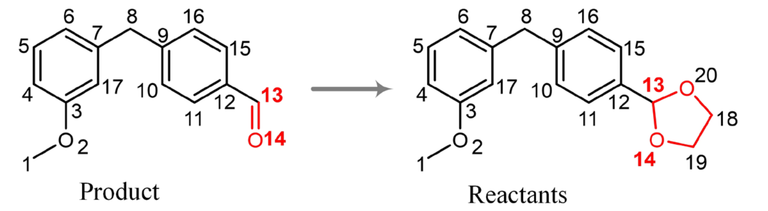


Incorrect edits sequence:

1. ('Change Bond', (1, 0))
2. Terminate

Edit atoms: [[13, 14]]

**c. Boric acid esterification**

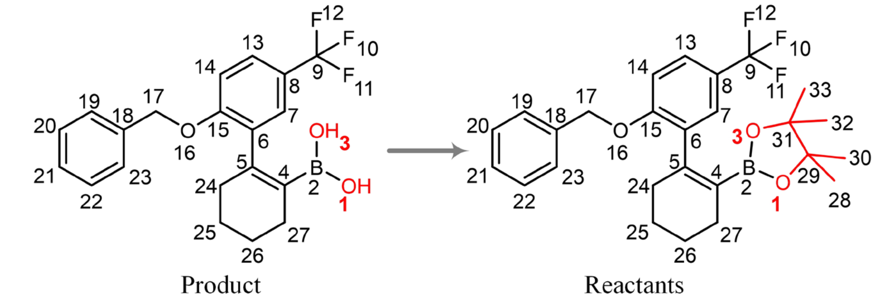


Incorrect edits sequence:

1. ('Change Atom', (0, 0))
2. ('Change Atom', (0, 0))
3. Terminate

Edit atoms: [[7, 8], 6, 8]

**Supplementary Fig. 2. Examples of incorrect graph edits sequence derived from retro-reactions. a** Amino protection reaction, **b** Aldehyde deprotection reaction, and **c** Boric acid esterification. The changes in the reaction are highlighted in red, and a common feature of these reactions is that the same leaving group needs to be added to more than one atom.

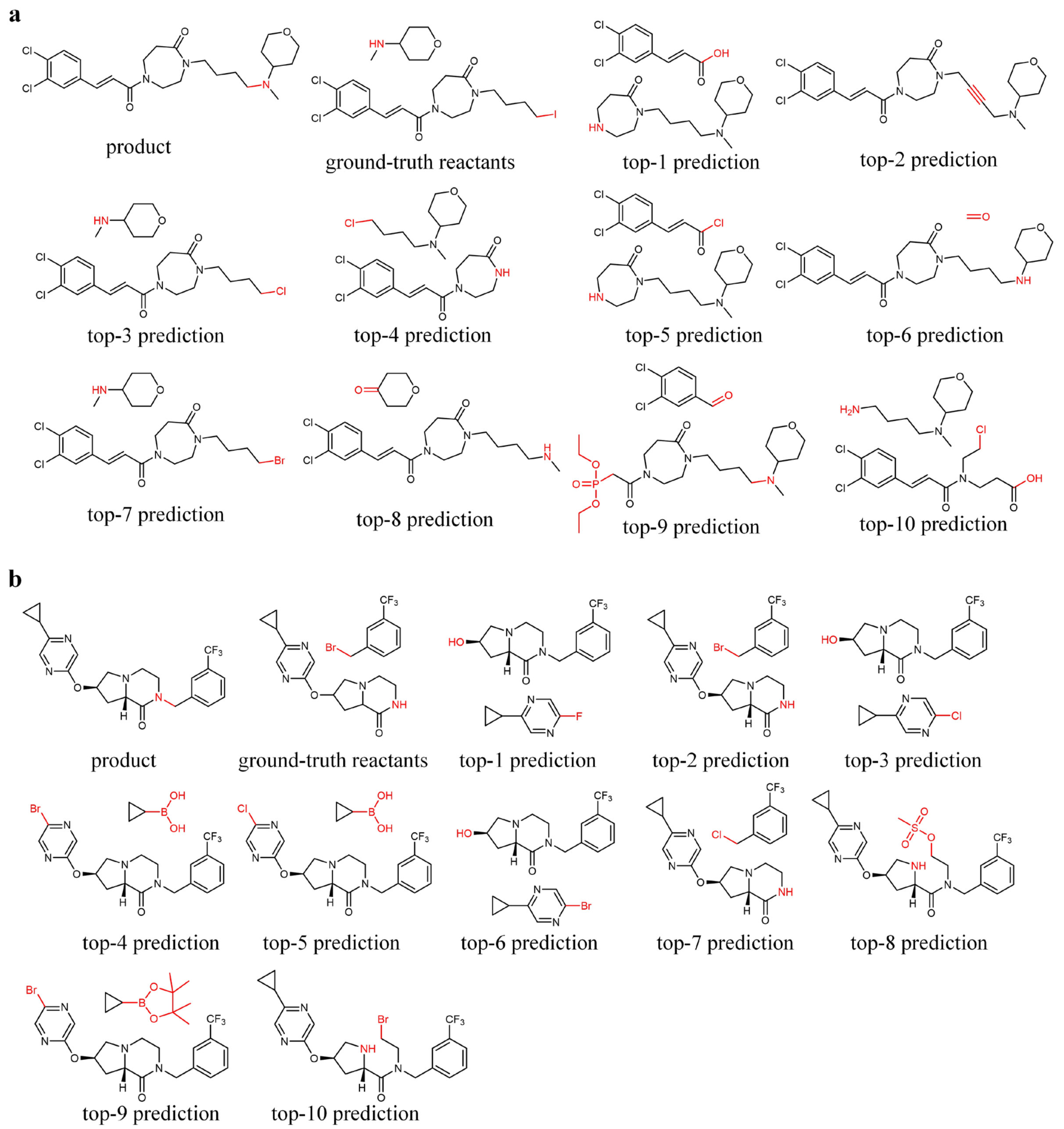


**Supplementary Fig. 3. Top-10 predictions by Graph2Edits for 2 random products from the USPTO-50k test set on which the predictions are different from the ground truth. a** The product can be yielded by different reaction types and Graph2Edits provides the options of replacing I with Cl and Br, **b** The ground-truth reactants is probably wrong and Graph2Edits correctly predicts the chiral reactants. The changes in the reaction are highlighted in red.

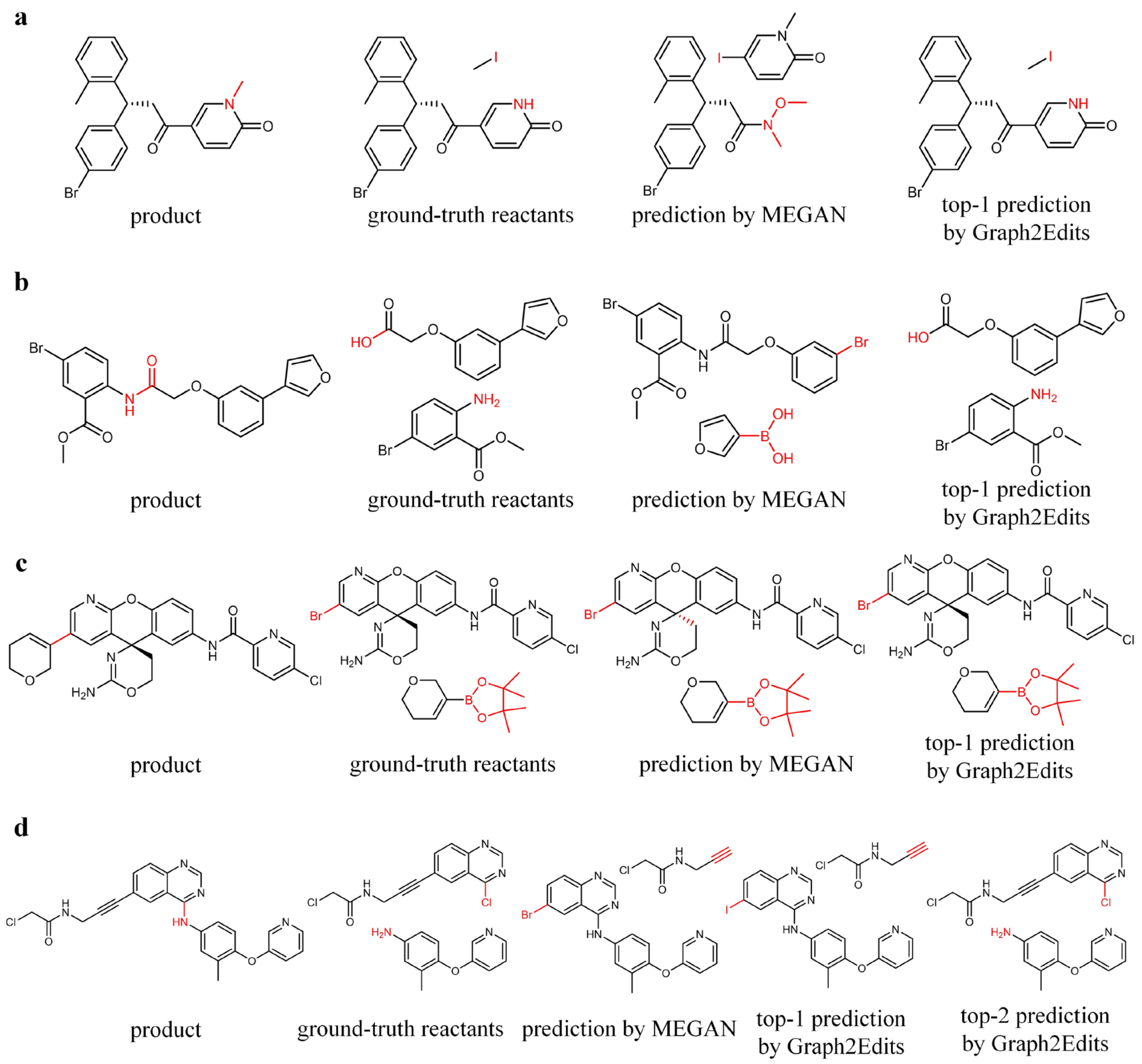


**Supplementary Fig. 4. Examples of comparison of predictions by MEGAN and Graph2Edits model.** The error categories in MEGAN predictions are **a** Only possible in multiple steps, **b** Low yield or side products, **c** Incorrect chirality, and **d** A reactive functional group ignored. The changes in the reaction are highlighted in red.

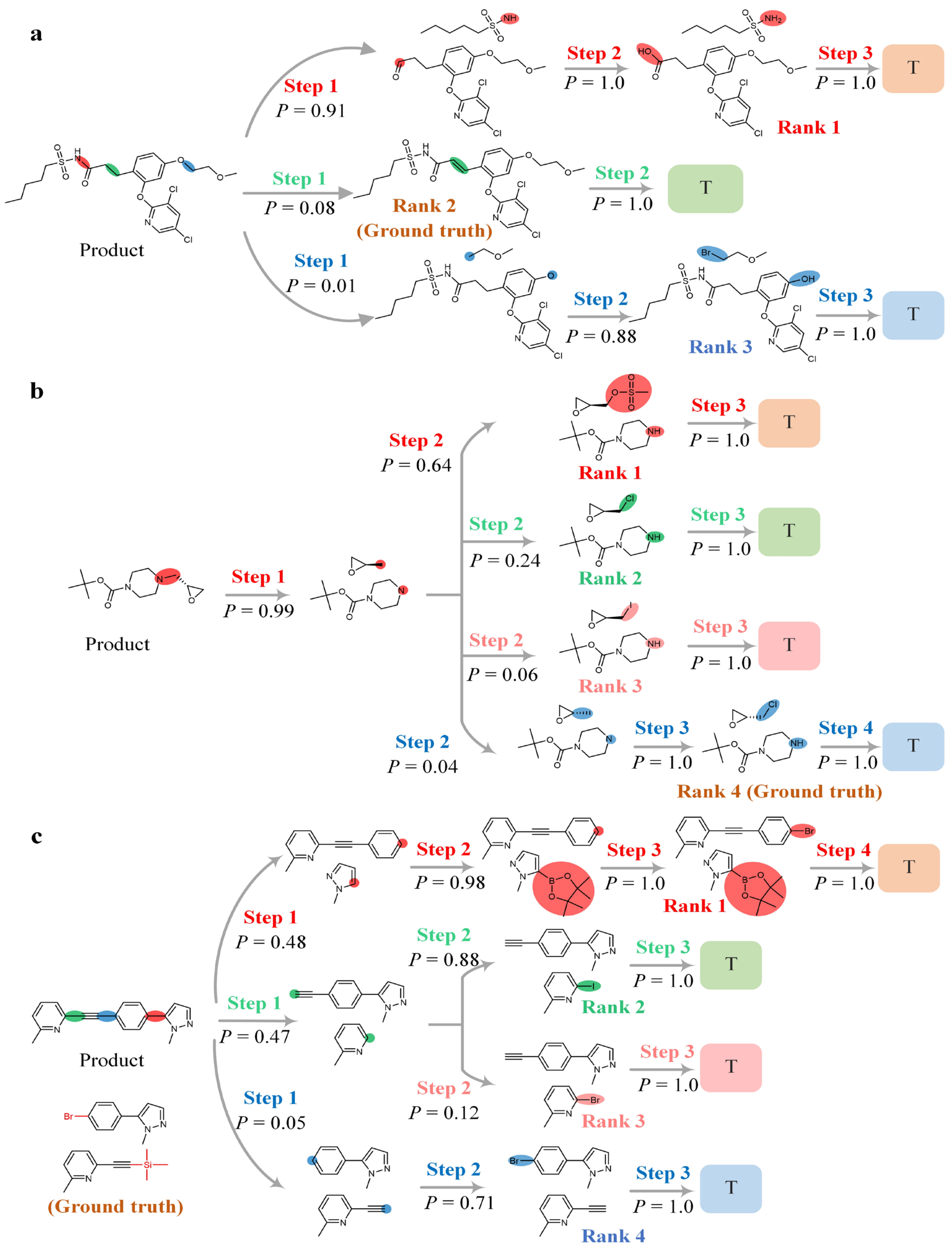


**Supplementary Fig. 5. More examples of retrosynthesis reasoning predictions by our model. a** Reduction reaction, **b** $S_N2$ Substitution reaction, **c** Silyl alkyne coupling reaction. The '*P*' is the probability of model prediction, and the 'T' represents a termination symbol.

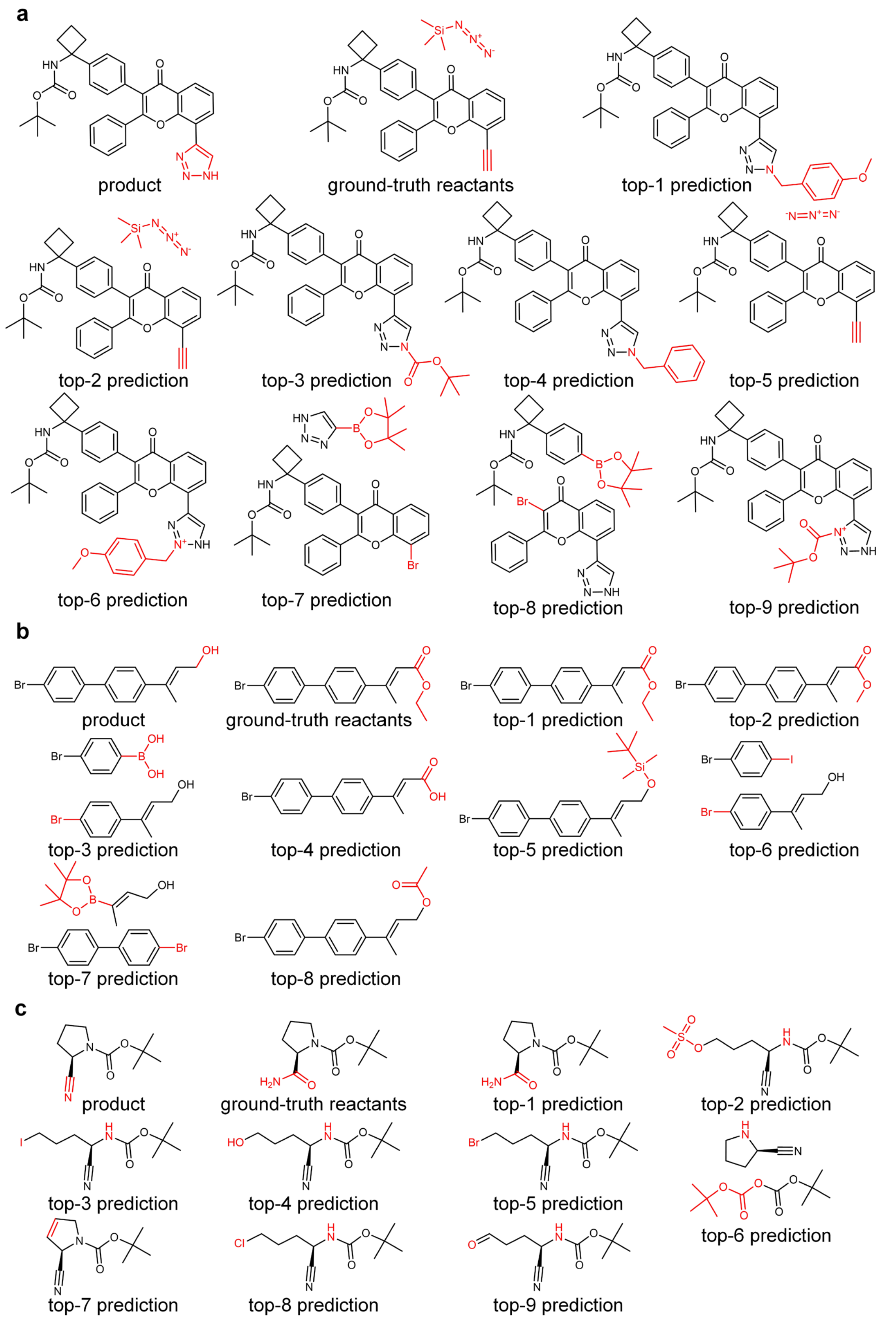


**Supplementary Fig. 6 Examples of predicted reactions by Graph2Edits. a** 1, 3-dipolar cycloaddition, **b** Reduction reaction, and **c** Dehydration reaction.

## Supplementary Tables

**Supplementary Data 1. Graph edits found in the USPTO-50k training set.**

| Number | Graph edits |
|---|---|
| 1 | ('Delete Bond', (None, None)) |
| 2 | ('Change Bond', (2, 0)) |
| 3 | ('Change Bond', (1, 0)) |
| 4 | ('Change Bond', (3, 0)) |
| 5 | ('Change Bond', (2, 3)) |
| 6 | ('Change Bond', (2, 2)) |
| 7 | ('Change Atom', (1, 0)) |
| 8 | ('Change Atom', (0, 0)) |
| 9 | ('Change Atom', (1, 1)) |
| 10 | ('Change Atom', (1, 2)) |
| 11 | ('Change Atom', (0, 2)) |
| 12 | ('Change Atom', (2, 0)) |
| 13 | ('Change Atom', (0, 1) |
| 14 | ('Attaching LG', '*C(=O)OCc1ccccc1') |
| 15 | ('Attaching LG', '*O') |
| 16 | ('Attaching LG', '*=O') |
| 17 | ('Attaching LG', '*Cl') |
| 18 | ('Attaching LG', '*Br') |
| 19 | ('Attaching LG', '*B(O)O') |
| 20 | ('Attaching LG', '*B1OC(C)(C)C(C)(C)O1') |
| 21 | ('Attaching LG', '*C(C)(C)C') |
| 22 | ('Attaching LG', '*Cc1ccc(OC)cc1') |
| 23 | ('Attaching LG', '*C') |
| 24 | ('Attaching LG', '*F') |
| 25 | ('Attaching LG', '*Cc1ccccc1') |
| 26 | ('Attaching LG', '*[Si](C)(C)C') |
| 27 | ('Attaching LG', '*=P(c1ccccc1)(c1ccccc1)c1ccccc1') |
| 28 | ('Attaching LG', '*OC(=O)OC(C)(C)C') |
| 29 | ('Attaching LG', '*I') |
| 30 | ('Attaching LG', '*[O-]') |
| 31 | ('Attaching LG', '*OCC') |
| 32 | ('Attaching LG', '*N1C(=O)CCC1=O') |
| 33 | ('Attaching LG', '*OS(=O)(=O)C(F)(F)F') |
| 34 | ('Attaching LG', '*OCC(F)(F)F') |
| 35 | ('Attaching LG', '*CC') |
| 36 | ('Attaching LG', '*OC') |
| 37 | ('Attaching LG', '*C(=O)OC(C)(C)C') |

| | |
|---|---|
| 38 | ('Attaching LG', '*N') |
| 39 | ('Attaching LG', '*Cl') |
| 40 | ('Attaching LG', '*[Si](C)(C)C(C)(C)C') |
| 41 | ('Attaching LG', '*OC(C)=O') |
| 42 | ('Attaching LG', '*C(C)=O') |
| 43 | ('Attaching LG', '*[P+](c1ccccc1)(c1ccccc1)c1ccccc1') |
| 44 | ('Attaching LG', '*[Sn](CCCC)(CCCC)CCCC') |
| 45 | ('Attaching LG', '*N(C)C') |
| 46 | ('Attaching LG', '*C(=O)OC') |
| 47 | ('Attaching LG', '*OS(C)(=O)=O') |
| 48 | ('Attaching LG', '*B1OCC(C)(C)CO1') |
| 49 | ('Attaching LG', '*[Mg+]') |
| 50 | ('Attaching LG', '*C(=O)c1ccccc1C(*)=O') |
| 51 | ('Attaching LG', '*OC(=O)c1cccc(Cl)c1') |
| 52 | ('Attaching LG', '*OC(C)(C)C') |
| 53 | ('Attaching LG', '*OC(=O)CCCCCCCCCCCCCCCCCCC') |
| 54 | ('Attaching LG', '*P(=O)(OCC)OCC') |
| 55 | ('Attaching LG', '*C(Cl)(Cl)Cl') |
| 56 | ('Attaching LG', '*OC(=O)C(F)(F)F') |
| 57 | ('Attaching LG', '*OCC(C)C') |
| 58 | ('Attaching LG', '*[Cu]') |
| 59 | ('Attaching LG', '*OS(=O)O') |
| 60 | ('Attaching LG', '*OC(=O)CC') |
| 61 | ('Attaching LG', '*B1OC(C)C(C)O1') |
| 62 | ('Attaching LG', '*N(C)OC') |
| 63 | ('Attaching LG', '*[Zn+]') |
| 64 | ('Attaching LG', '*C(Br)(Br)Br') |
| 65 | ('Attaching LG', '*C(=O)CCl') |
| 66 | ('Attaching LG', '*OC=O') |
| 67 | ('Attaching LG', '*C1CCCCO1') |
| 68 | ('Attaching LG', '*=[N+]=[N-]') |
| 69 | ('Attaching LG', '*C(=O)C(C)C') |
| 70 | ('Attaching LG', '*[S+](C)(C)=O') |
| 71 | ('Attaching LG', '*=C') |
| 72 | ('Attaching LG', '*C(=O)CCCCCCCCCCCCCCC') |
| 73 | ('Attaching LG', '*OC(=O)OCC') |
| 74 | ('Attaching LG', '*C(Br)Br') |
| 75 | ('Attaching LG', '*OCC(Cl)(Cl)Cl') |
| 76 | ('Attaching LG', '*C(=O)C(F)(F)F') |
| 77 | ('Attaching LG', '*[Sn](CC)(CC)CC') |
| 78 | ('Attaching LG', '*[S+](C)C') |
| 79 | ('Attaching LG', '*C(=O)CCC') |
| 80 | ('Attaching LG', '*[Si](C(C)C)(C(C)C)C(C)C') |

| | |
|---|---|
| 81 | ('Attaching LG', '*COC') |
| 82 | ('Attaching LG', '*[Sn](C)(C)C') |
| 83 | ('Attaching LG', '*C(=O)c1ccccc1') |
| 84 | ('Attaching LG', '*C(=O)OCC') |
| 85 | ('Attaching LG', '*OCCC') |
| 86 | ('Attaching LG', '*OC(=O)C(C)(C)C') |
| 87 | ('Attaching LG', '*C(=O)OCC1c2ccccc2-c2ccccc21') |
| 88 | ('Attaching LG', '*OC(C)Cl') |
| 89 | ('Attaching LG', '*[P+](C)(C)C') |
| 90 | ('Attaching LG', '*OCCCCCCCCCCCC') |
| 91 | ('Attaching LG', '*n1ccnc1') |
| 92 | ('Attaching LG', '*B1OB(C)OB(C)O1') |
| 93 | ('Attaching LG', '*B1OCCN(c2ccccc2)CCO1') |
| 94 | ('Attaching LG', '*B1OC(=O)CN(C)CC(=O)O1') |
| 95 | ('Attaching LG', '*B1OB(C=C)OB(C=C)O1') |
| 96 | ('Attaching LG', '*C(=O)CC') |
| 97 | ('Attaching LG', '*C(c1ccccc1)c1ccccc1') |
| 98 | ('Attaching LG', '*OC(=O)CCCCCCCC') |
| 99 | ('Attaching LG', '*OC(=O)CCl') |
| 100 | ('Attaching LG', '*OC(=O)C(F)(F)Cl') |
| 101 | ('Attaching LG', '*OC(=O)OCc1ccccc1') |
| 102 | ('Attaching LG', '*P(=O)(OC)OC') |
| 103 | ('Attaching LG', '*OC(=O)C(C)C') |
| 104 | ('Attaching LG', '*C(=O)c1ccc([N+](=O)[O-])cc1') |
| 105 | ('Attaching LG', '*OC(Cl)(Cl)Cl') |
| 106 | ('Attaching LG', '*[Zn]Br') |
| 107 | ('Attaching LG', '*[Si](C)(C)C(C)(C)C(C)C') |
| 108 | ('Attaching LG', '*OCc1ccccc1') |
| 109 | ('Attaching LG', '*B1OC(C)CC(C)(C)O1') |
| 110 | ('Attaching LG', '*B(OC(C)C)OC(C)C') |
| 111 | ('Attaching LG', '*OCCCC') |
| 112 | ('Attaching LG', '*OC(C)C(C)=O') |
| 113 | ('Attaching LG', '*=N[Si](C)(C)C') |
| 114 | ('Attaching LG', '*OC1NCC(=C)O1') |
| 115 | ('Attaching LG', '*=S') |
| 116 | ('Attaching LG', '*OC(=O)c1ccccc1Cl') |
| 117 | ('Attaching LG', '*C(=O)N(C)C') |
| 118 | ('Attaching LG', '*=C(Cl)CCCC') |
| 119 | ('Attaching LG', '*Cc1cc([N+](=O)[O-])ccc1O') |
| 120 | ('Attaching LG', '*[Si](CC)(CC)CC') |
| 121 | ('Attaching LG', '*B1OCCO1') |
| 122 | ('Attaching LG', '*OC(=O)c1ccccc1') |
| 123 | ('Attaching LG', '*[Mg]Br') |

| | |
|---|---|
| 124 | ('Attaching LG', '*OC(=O)C(F)F') |
| 125 | ('Attaching LG', '*CCCC') |
| 126 | ('Attaching LG', '*OC(=O)CCCC') |
| 127 | ('Attaching LG', '*C(C)C') |
| 128 | ('Attaching LG', '*C(=O)OCC=C') |
| 129 | ('Attaching LG', '*C(=O)[C@H](C)c1ccc2cc(OC)ccc2c1') |
| 130 | ('Attaching LG', '*C(=O)C(C)(C)C') |
| 131 | ('Attaching LG', '*OC(=O)C(=C)C') |
| 132 | ('Attaching LG', '*[N+](C)(C)C') |
| 133 | ('Attaching LG', '*C(=O)[C@@H](OC(C)=O)c1ccccc1') |
| 134 | ('Attaching LG', '*[Zn]I') |
| 135 | ('Attaching LG', '*S(C)(=O)=O') |
| 136 | ('Attaching LG', '*OC(=O)CCCCC') |
| 137 | ('Attaching LG', '*B1OC=CC(C(=O)O)O1') |
| 138 | ('Attaching LG', '*OC(=O)C1CC1') |
| 139 | ('Attaching LG', '*OCC(C)(C)NS(=O)(=O)C(F)(F)F') |
| 140 | ('Attaching LG', '*OC(=O)CBr') |
| 141 | ('Attaching LG', '*C(=O)/C=C/C=C/C=C/C=C/C(=O)O') |
| 142 | ('Attaching LG', '*B1OB(c2ccc(F)cc2)OB(c2ccc(F)cc2)O1') |
| 143 | ('Attaching LG', '*OC(=O)CCC') |
| 144 | ('Attaching LG', '*OC(=O)C(=NOC)c1csc(NC(c2ccccc2)(c2ccccc2)c2ccccc2)n1') |
| 145 | ('Attaching LG', '*C(=O)c1ccc(OCCCCCCC)cc1') |
| 146 | ('Attaching LG', '*OC(=O)/C=C\\n1cnc(-c2cc(OC)cc(C(F)(F)F)c2)n1') |
| 147 | ('Attaching LG', '*C(=O)c1ccc(OC)cc1') |
| 148 | ('Attaching LG', '*P(=O)(OCC(F)(F)F)OCC(F)(F)F') |
| 149 | ('Attaching LG', '*C(=O)CCCC') |
| 150 | ('Attaching LG', '*OC(=O)CI') |
| 151 | ('Attaching LG', '*=NS(=O)(=O)C(F)(F)F') |
| 152 | ('Attaching LG', '*OCc1ccc([N+](=O)[O-])cc1') |
| 153 | ('Attaching LG', '*C(=O)C1(C)CCCCC1') |
| 154 | ('Attaching LG', '*C(=O)CCCCBr') |
| 155 | ('Attaching LG', '*C(=O)CCC(=O)OC') |
| 156 | ('Attaching LG', '*C(=O)c1cccc(Cl)c1') |
| 157 | ('Attaching LG', '*B1OCCCO1') |
| 158 | ('Attaching LG', '*OC(C)C') |
| 159 | 'Terminate' |

**Supplementary Table 2. Top-k exact match accuracy of the proposed Graph2Edits and baselines on USPTO-full dataset.**

| Category | Model | Top-k accuracy (%) | | | |
|---|---|---|---|---|---|
| | | k = 1 | 3 | 5 | 10 |
| Template-Based | Retrosim | 32.8 | - | - | 56.1 |
| | Neuralsym | 35.8 | - | - | 60.8 |
| | GLN | 39.3 | - | - | 63.7 |
| Template-Free | Aug.Transformer | 46.2 | - | - | 73.3 |
| | GTA | 46.6 | - | - | 70.4 |
| | Graph2SMILES | 45.7 | - | - | 63.4 |
| Semi-Template-Based | RetroPrime | 44.1 | 59.1 | 62.8 | 68.5 |
| | MEGAN | 33.6 | - | - | 63.9 |
| | Graph2Edits (D-MPNN) | 44.0 | 60.9 | 66.8 | 72.5 |

Graph2Edits (D-MPNN) uses the directed message passing neural network (D-MPNN) as graph encoder.

**Supplementary Table 3. Top-1 retrosynthesis prediction by Graph2Edits for 30 random target products from USPTO-50k test set on which the prediction is different from the ground truth.**

| | Product | Ground-truth reactants | Top-1 prediction |
|---|---|---|---|
| 1 | 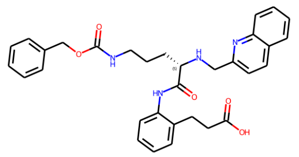 | 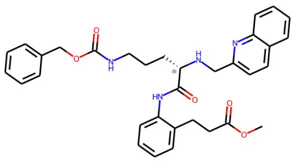 | 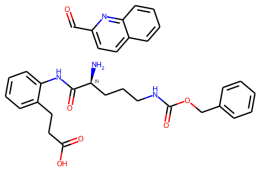 |
| 2 | 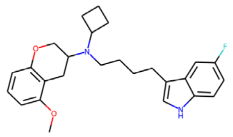 | 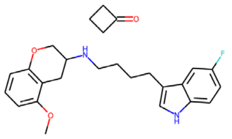 | 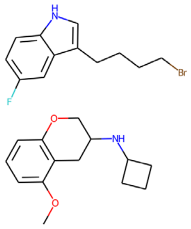 |
| 3 | 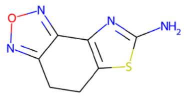 | 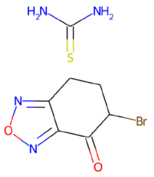 | 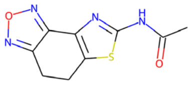 |
| 4 | 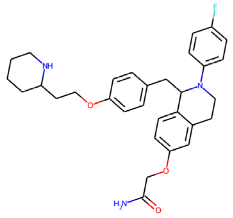 | 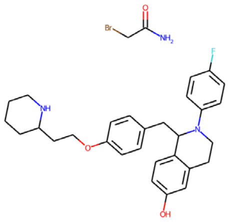 | 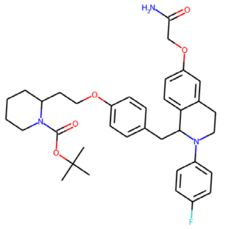 |
| 5 | 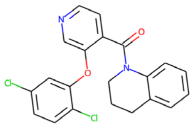 | 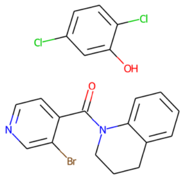 | 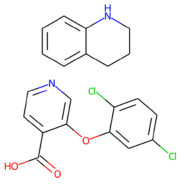 |
| 6 | 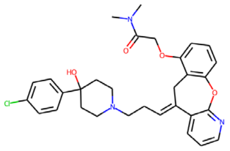 | 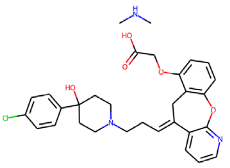 | 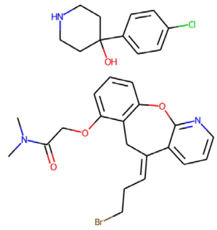 |
| 7 | 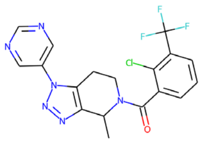 | 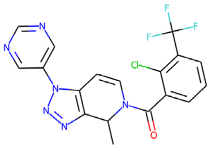 | 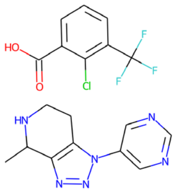 |

| | | | |
|---|---|---|---|
| 8 | 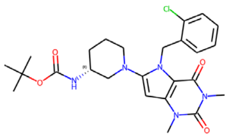 | 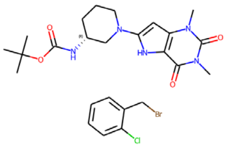 | 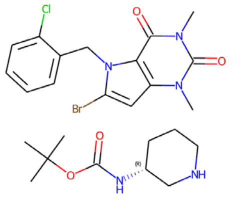 |
| 9 | 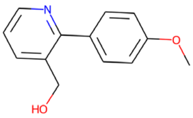 | 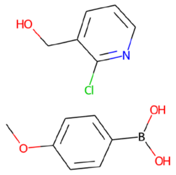 | 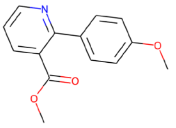 |
| 10 | 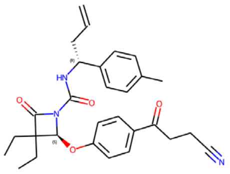 | 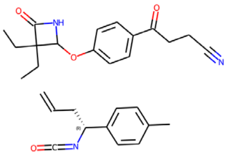 | 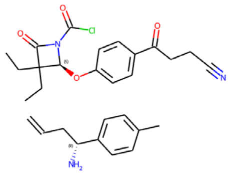 |
| 11 | 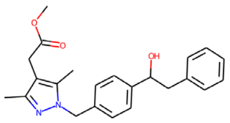 | 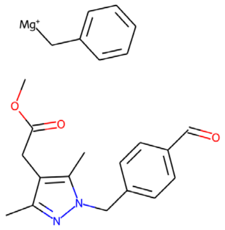 | 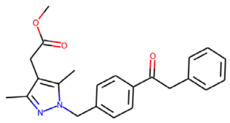 |
| 12 | 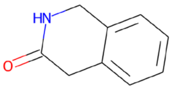 | 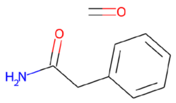 | 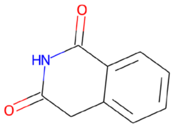 |
| 13 | 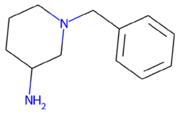 | 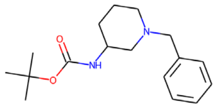 | 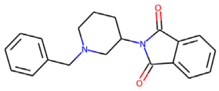 |
| 14 | 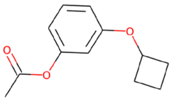 | 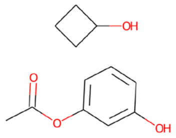 | 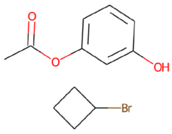 |
| 15 | 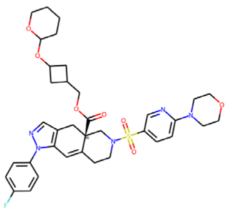 | 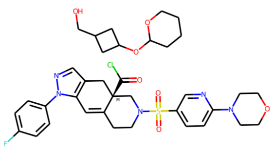 | 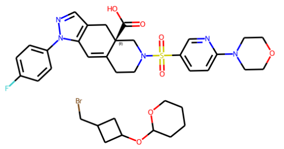 |

| | | | |
|---|---|---|---|
| 16 | 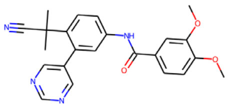 | 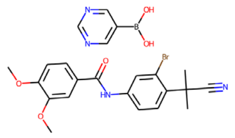 | 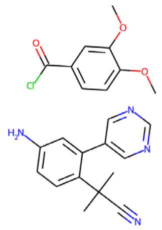 |
| 17 | 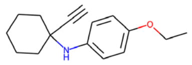 | 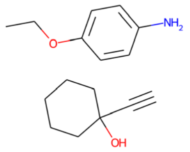 | 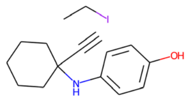 |
| 18 | 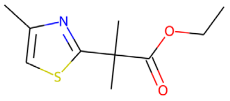 | 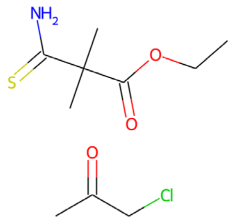 | 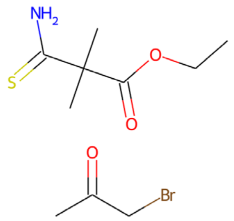 |
| 19 | 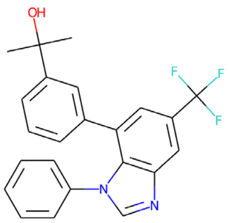 | 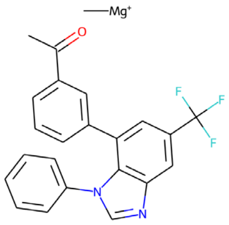 | 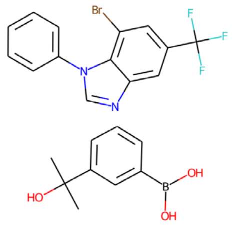 |
| 20 | 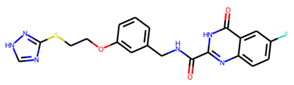 | 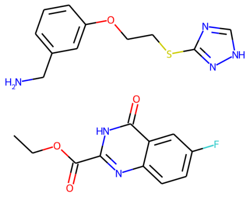 | 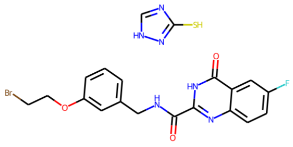 |
| 21 | 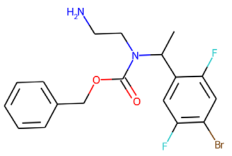 | 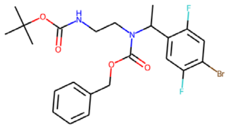 | 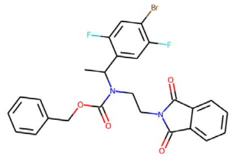 |
| 22 | 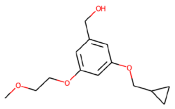 | 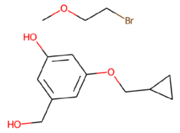 | 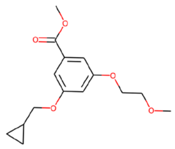 |
| 23 | 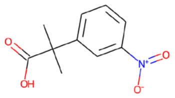 | 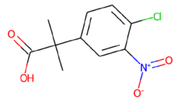 | 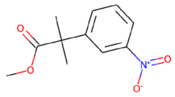 |

| | | | |
|---|---|---|---|
| 24 | 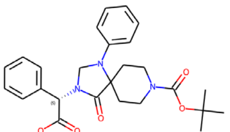 | 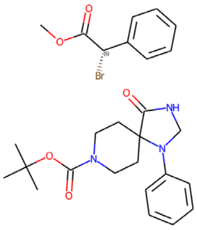 | 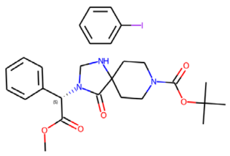 |
| 25 | 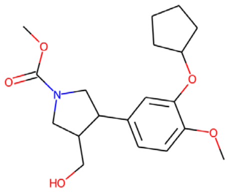 | 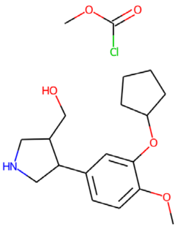 | 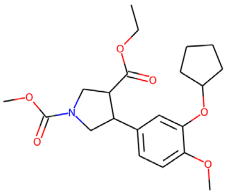 |
| 26 | 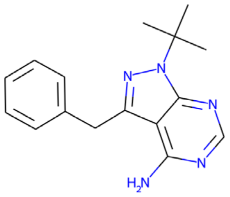 | 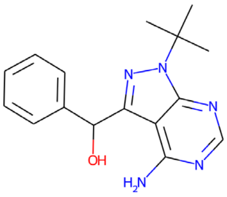 | 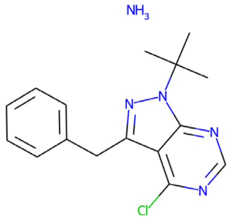 |
| 27 | 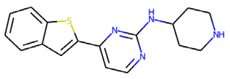 | 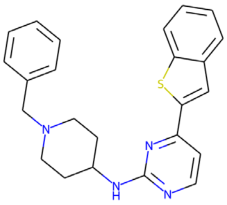 | 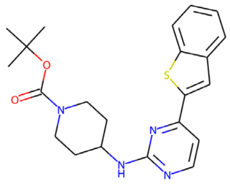 |
| 28 | 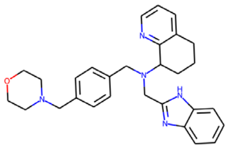 | 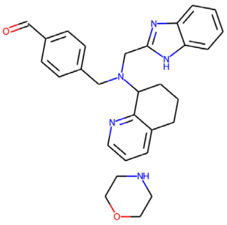 | 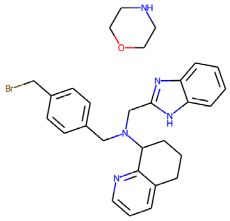 |
| 29 | 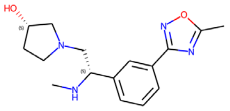 | 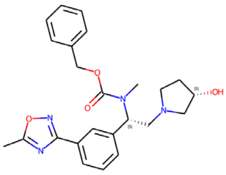 | 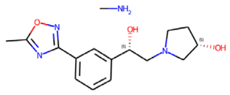 |
| 30 | 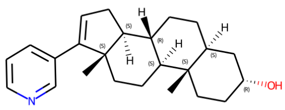 | 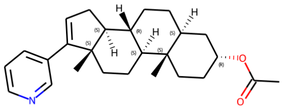 | 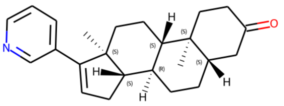 |

**Supplementary Table 4. Comparison of invalid rates among seq2seq, SCROP, and our model Graph2Edits for different beam sizes on USPTO-50k dataset.**

| **Model** | **Top-k invalid rate (%)** | | | |
|---|---|---|---|---|
| | **k = 1** | **3** | **5** | **10** |
| seq2seq | 12.2 | 15.3 | 18.4 | 22.0 |
| SCROP | 0.7 | 1.4 | 1.8 | 2.3 |
| Graph2Edits | 0 | 0.71 | 0.86 | 0.87 |

**Supplementary Table 5. Top-1 retrosynthesis prediction by Graph2Edits for 30 random reactions with stereochemistry change from USPTO-50k test set.**

| | Product | Ground-truth reactants | Top-1 prediction |
|---|---|---|---|
| 1 | 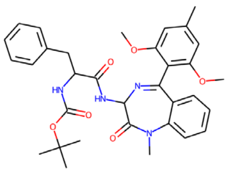 | 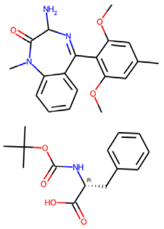 | 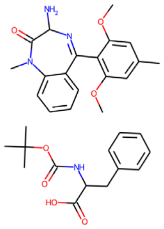 |
| 2 | 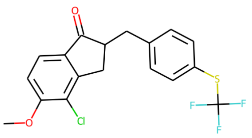 | 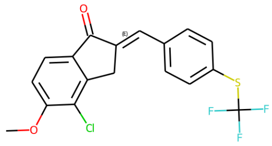 | 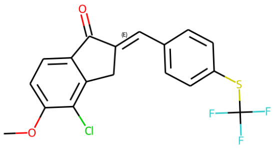 |
| 3 | 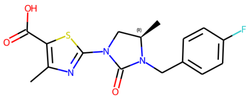 | 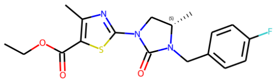 | 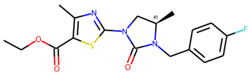 |
| 4 | 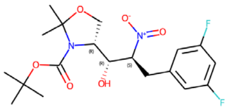 | 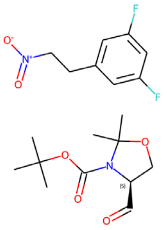 | 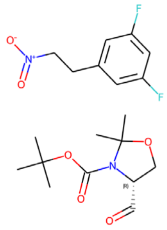 |
| 5 | 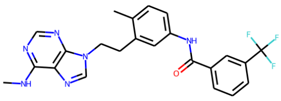 | 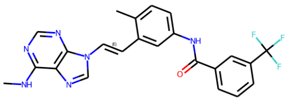 | 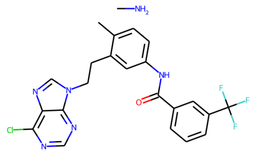 |
| 6 | 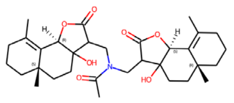 | 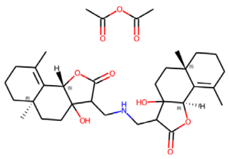 | 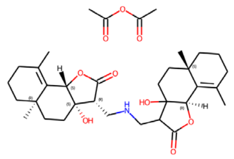 |
| 7 | 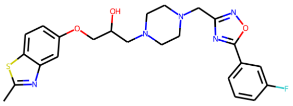 | 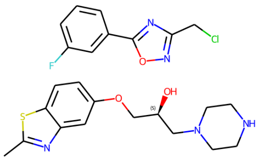 | 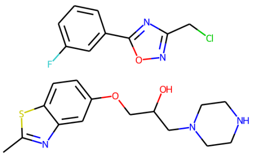 |

| | | | |
|---|---|---|---|
| 8 | 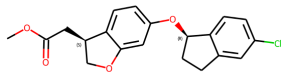 | 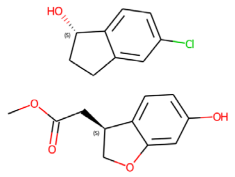 | 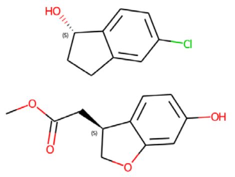 |
| 9 | 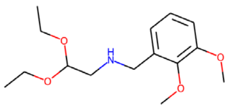 | 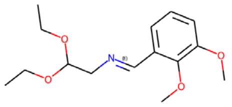 | 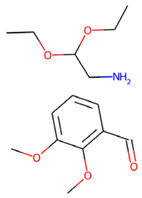 |
| 10 | 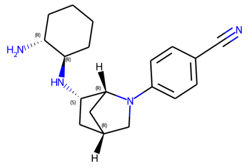 | 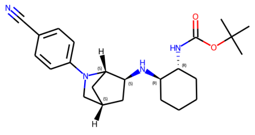 | 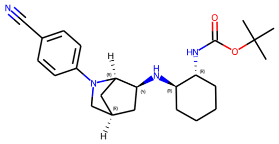 |
| 11 | 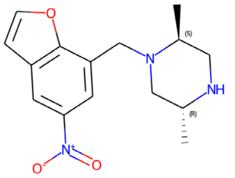 | 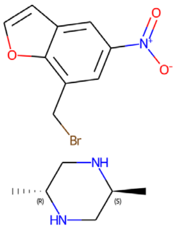 | 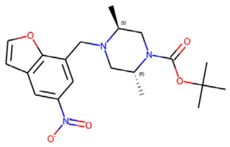 |
| 12 | 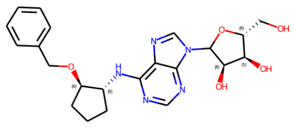 | 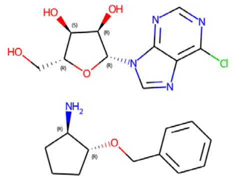 | 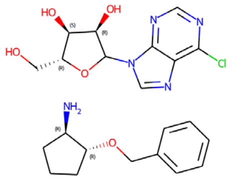 |
| 13 | 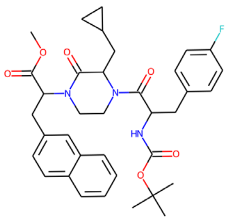 | 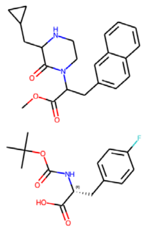 | 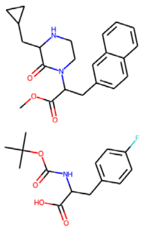 |
| 14 | 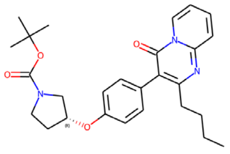 | 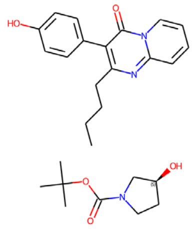 | 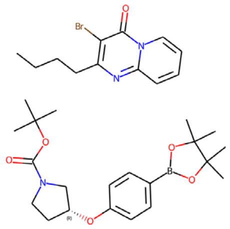 |
| 15 | 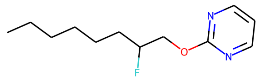 | 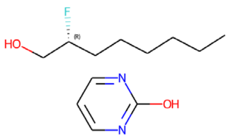 | 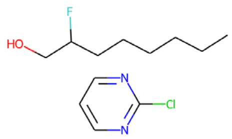 |

| | | | |
|---|---|---|---|
| 16 | 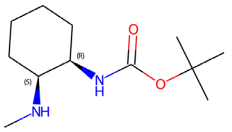 | 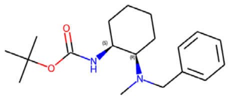 | 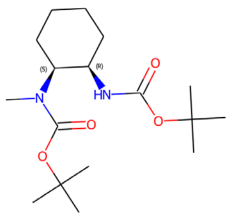 |
| 17 | 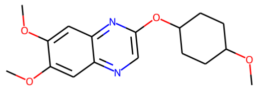 | 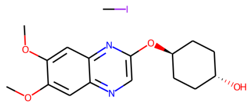 | 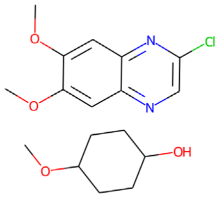 |
| 18 | 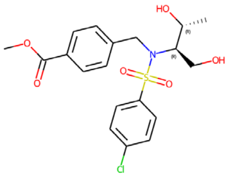 | 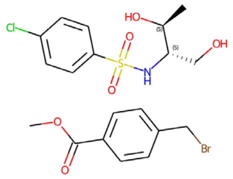 | 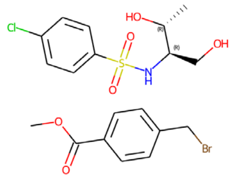 |
| 19 | 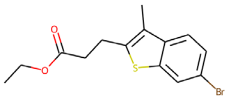 | 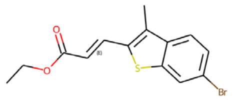 | 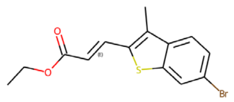 |
| 20 | 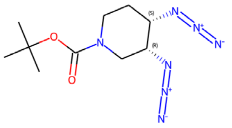 | 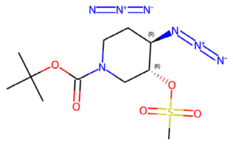 | 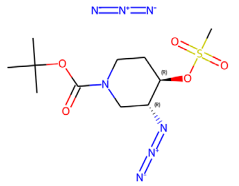 |
| 21 | 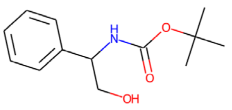 | 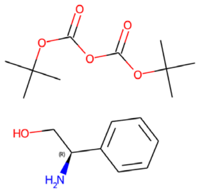 | 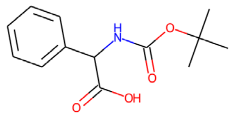 |
| 22 | 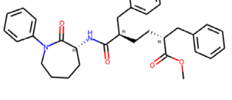 | 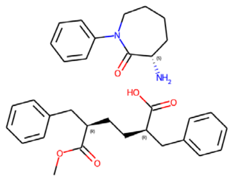 | 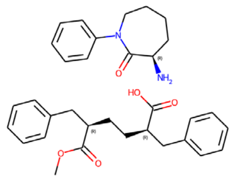 |
| 23 | 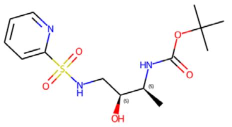 | 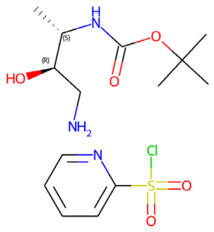 | 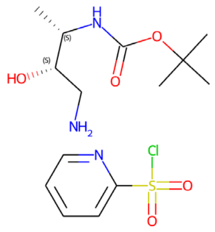 |

| | | | |
|---|---|---|---|
| 24 | 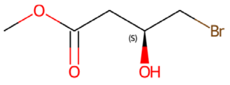 | 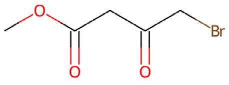 | 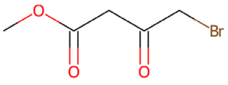 |
| 25 | 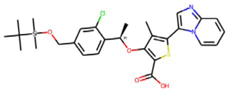 | 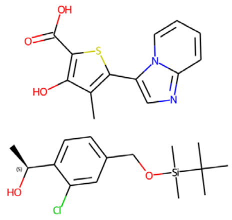 | 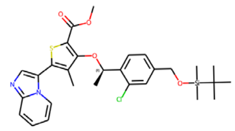 |
| 26 | 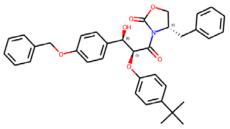 | 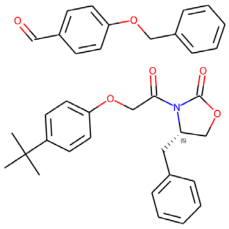 | 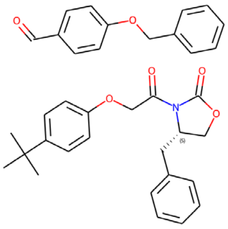 |
| 27 | 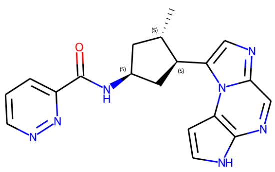 | 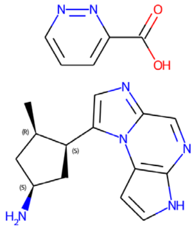 | 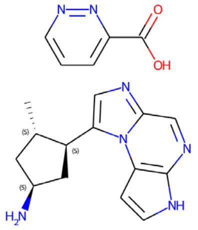 |
| 28 | 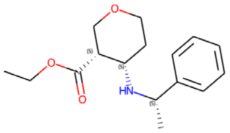 | 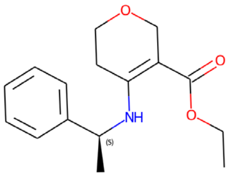 | 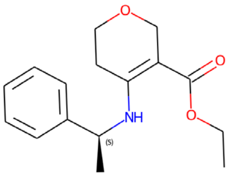 |
| 29 | 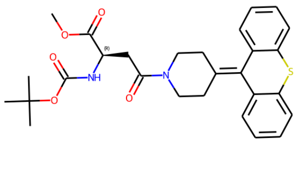 | 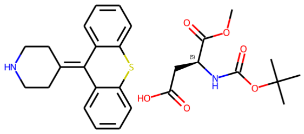 | 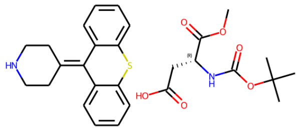 |
| 30 | 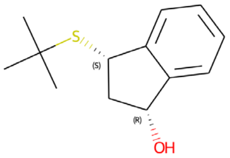 | 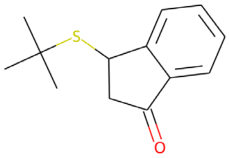 | 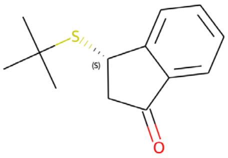 |

**Supplementary Table 6. Initialization of atom features.**

| Name | Description | Dimension |
|---|---|---|
| Atom type | [C, N, O, F, Cl, Br, I, etc.] | 48 (one-hot) |
| Degree | Number of heavy atom neighbors [0, 1, 2, 3, 4, 5, 6, 7, 8, 9] | 10 (one-hot) |
| Formal charge | [-1, -2, 1, 2, 0] | 5 (one-hot) |
| Valence | [0, 1, 2, 3, 4, 5, 6,] | 7 (one-hot) |
| Number of hydrogens | [0, 1, 2, 3, 4] | 5 (one-hot) |
| Chirality | Unspecified, tetrahedral CW/CCW, or other. | 4 (one-hot) |
| Hybridization | [sp, sp2, sp3, sp3d, sp3d2] | 5 (one-hot) |
| Aromatic | Whether the atom is part of an aromatic system | 1 |

**Supplementary Table 7. Initialization of bond features.**

| Name | Description | Dimension |
|---|---|---|
| Bond type | [single, double, triple, aromatic] | 4 (one-hot) |
| Bond stereo | [None, any, E/Z or cis/trans] | 6 (one-hot) |
| Conjugated | Whether the bond is part of a conjugated system | 1 |
| Ring | Whether the bond is part of a ring | 1 |

## Supplementary Notes

**Evaluation of invalid rates.** The template-free approaches can output grammatically invalid predictions and our model Graph2Edits can also generate "invalid edits" that do not bring to a valid chemical structure. To evaluate the invalid rates, we calculated the top-*k* percentage of invalid smiles generated by Graph2Edits and compared it with the template-free models seq2seq[1] and SCROP[2]. As shown in Supplementary Table 4, the top-1 predictions generated by our model are all valid chemical structures, and only 0.71% and 0.87% of the top-3 and top-10 predictions are grammatically invalid, which is significantly better than those by the template-free models seq2seq and SCROP (used the syntax corrector).

## Supplementary References


1 Liu, B. et al. Retrosynthetic reaction prediction using neural sequence-to-sequence models. *ACS central science* **3**, 1103-1113 (2017).

2 Zheng, S., Rao, J., Zhang, Z., Xu, J. & Yang, Y. Predicting retrosynthetic reactions using self-corrected transformer neural networks. *Journal of chemical information and modeling* **60**, 47-55 (2019).
