# Supplementary material for: Retrosynthesis prediction using an end-to-end graph generative architecture for molecular graph editing
Source: Nat Commun. 2023 May 25;14:3009. doi: 10.1038/s41467-023-38851-5 (PMC10209957; doi:10.1038/s41467-023-38851-5)
Supplement: Supplementary file 3 — Description of Additional Supplementary Files [file 41467_2023_38851_MOESM3_ESM.pdf]

Description of Additional Supplementary Files

File Name: Supplementary Data 1

Description: Details of the graph edits found in the USPTO-50k training set. This table provides all graph edits were derived from the training set of USPTO-50k dataset, including 6 bond edits, 152 atom edits (7 Change Atom and 145 Attach LG), and a termination symbol.

File Name: Supplementary Data 2

Description: The performance effect of edits sequence length. This table provides the source data of **Figure 3**. This table presents the quantities and top-10 exact match accuracy of different edits sequence length reaction categories in the USPTO-50k test set.

File Name: Supplementary Data 3

Description: The cluster results on USPTO-50k test set based on predicted reactants similarities. This table provides the source data of **Figure 5**. This table provides the cluster classes, the the predicted reactants similarities, and the reaction quantities on USPTO-50k test set. The lower similarity indicates the higher diversity of model predicted results.
